# Identification of Immune-Related Risk Characteristics and Prognostic Value of Immunophenotyping in TNBC

**DOI:** 10.3389/fgene.2021.730442

**Published:** 2021-10-29

**Authors:** Jiarong Yi, Zeyu Shuang, Wenjing Zhong, Haoming Wu, Jikun Feng, Xiazi Zouxu, Xinjian Huang, Siqi Li, Xi Wang

**Affiliations:** Department of Breast Oncology, Sun Yat-sen University Cancer Center, The State Key Laboratory of Oncology in South China, Collaborative Innovation Center for Cancer Medicine, Guangzhou, China

**Keywords:** TNBC, TCGA, GEO, immunophenotyping, prognosis

## Abstract

**Background:** Triple-negative breast cancer (TNBC) is not sensitive to targeted therapy with HER-2 monoclonal antibody and endocrine therapy due to lack of ER, PR, and HER-2 receptors. TNBC is a breast cancer subtype with the worst prognosis and the highest mortality rate compared with other subtypes.

**Materials and Methods:** Breast cancer-related data were retrieved from *The Cancer Genome Atlas* (TCGA) database, and 116 cases of triple-negative breast cancer were identified from the data. GSE31519 dataset was retrieved from *Gene Expression Omnibus* (GEO) database, comprising a total of 68 cases with TNBC. Survival analysis was performed based on immune score, infiltration score and mutation score to explore differences in prognosis of different immune types. Analysis of differentially expressed genes was conducted and GSEA analysis based on these genes was conducted to explore the potential mechanism.

**Results:** The findings showed that comprehensive immune typing is highly effective and accurate in assessing prognosis of TNBC patients. Analysis showed that MMP9, CXCL9, CXCL10, CXCL11 and CD7 are key genes that may affect immune typing of TNBC patients and play an important role in prediction of prognosis in TNBC patients.

**Conclusion:** The current study presents an evaluation system based on immunophenotyping, which provides a more accurate prognostic evaluation tool for TNBC patients. Differentially expressed genes can be targeted to improve treatment of TNBC.

## Introduction

Breast cancer has the highest incidence of cancer cases in women worldwide. Incidence of breast cancer is 11.7% and the mortality rate is 6.9%, thus it poses a significant health burden globally (Sung et al., 2021). Breast cancer is grouped into several subtypes based on molecular characteristics including: estrogen receptor positive and progesterone receptor positive (luminal A, luminal B), HER2 overexpression (HER2+), and triple-negative breast cancer (TNBC) (Chodosh, 2011). Individualized treatment plans have been explored for the different subtypes (Keshtgar et al., 2010). TNBC is a subtype of breast cancer in which estrogen receptor (ER) and progesterone receptor (PR) are not present, and HER2 is not expressed, and it accounts for approximately 15% in breast cancer cases. TNBC is not sensitive to targeted therapy with HER-2 receptor monoclonal antibody and endocrine therapy owing to the lack of these receptors. Although various treatments have been developed, more than 70% of TNBC patients present with recurrence and relapse within 3 years after surgical resection resulting in poor prognosis (Cronin et al., 2018; Huynh et al., 2020; Sharma, 2016).

Previous studies have explored classification strategies for cancer immunotyping, including infiltration score, immune score and mutation score (Li et al., 2017; Chen et al., 2020; Zhao et al., 2021). Immune score and infiltration score classification strategy has been used in lung cancer, urothelial cell carcinoma, bladder cancer (Fu et al., 2018; Tan et al., 2020; Zeng et al., 2020). However, studies have not explored classification of TNBC and breast cancer using these scores. In the current study, breast cancer-related data were retrieved from TCGA database, and a total of 116 cases of TNBC patients were identified. In addition, GSE31519 dataset was retrieved from GEO database comprising a total of 68 cases of TNBC patients. Comprehensive analysis of the two datasets was performed to explore the prognosis of immune score, infiltration score, and mutation score by survival analysis. Moreover, key genes were identified and their roles in predicting prognosis were explored through differential analysis. Potential pathways implicated in mechanism of differentially expressed genes were predicted through GSEA. An evaluation system based on immunophenotyping for use as an accurate prognostic evaluation tool for TNBC patients was developed. Differentially expressed genes can also be a targeted to develop effective TNBC therapy.

## Materials and Methods

**FIGURE 1:**
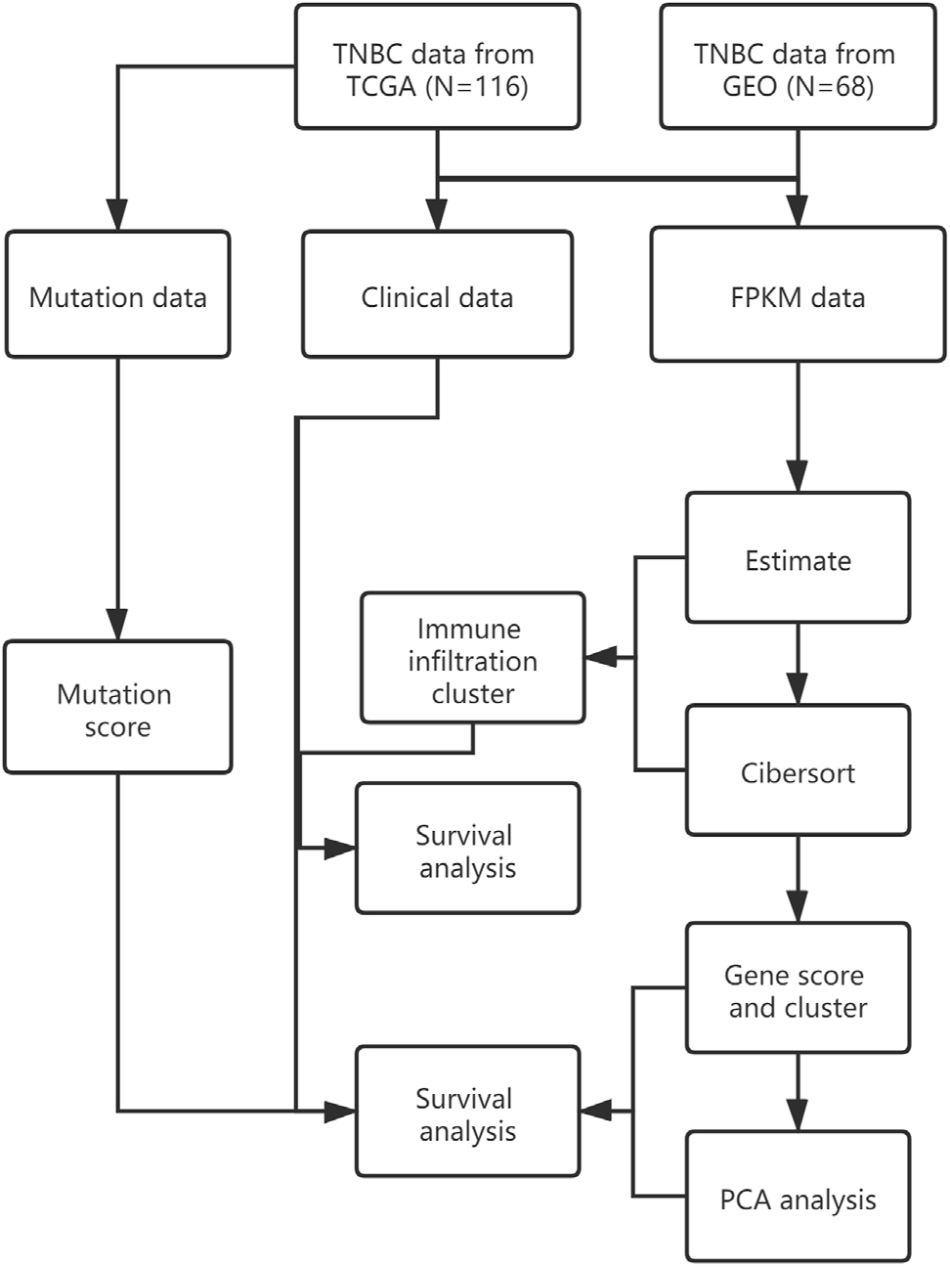
Research design.

The research design for the current study is presented in Figure 1.

### Gene Expression Dataset

TNBC dataset used in the current study comprised data retrieved from TCGA database and GEO database. TCGA dataset comprised basic information, gene expression profiles and prognostic information retrieved from TCGA database. The study only screened out patients who had been diagnosed with TNBC with confirmed pathology and clinical information. Patients with insufficient or missing data such as age, TNM staging, and OS were excluded. Information for a total of 116 patients was retrieved. GEO data was retrieved from GEO database by searching keywords “TNBC” and “survival,” and similar inclusion criteria that of TCGA data was used. GSE31519 dataset comprising information of 68 patients was retrieved from GEO database.

### Analysis of Immune Infiltration

Immune infiltration in TNBC patients was analyzed by ESTIMATE and CIBERSORT tools. ESTIMATE score was determined using ESTIMATE R package. The immune infiltration score for all patients, including stromal score and immune score were determined by comparing FPKM (Fragments Per Kilobase of exon model per Million mapped fragments) data with the standard information from the R package (Jia et al., 2018; Zhou et al., 2020). CIBERSORT score was determined using CIBERSORT R package. Information on 22 immune cells available in the databases on 547 immune-related markers in TNBC patients was retrieved. The relative scores of immune cells and relative proportion of immune cells was determined using CIBERSORT R package (Gentles et al., 2015; Newman et al., 2015).

### Immunophenotyping

An immune cluster was generated using the ConsensusClusterPlus R package based on CIBERSORT scores. The cluster parameter was set at 9 and the type parameter was set as 3. Survival analysis was performed using survival R package and survminer R package based on the results from immune cluster analysis and the clinical data of TCGA and GEO. Survival and immune infiltration analysis indicates the value of immunophenotyping in triple-negative breast cancer. A heat map was generated using pheatmap R package to show differences in different immunophenotypes in patients. Differentially expressed genes in the different immunophenotypes were explored using the limma R package to explored potential targets for regulating immune responses in TNBC patients (Shi et al., 2020).

### Genotyping

Transcriptome data retrieved from TCGA and GEO databases and the differentially expressed genes were genotyped using ConsensusClusterPlus R package and the limma R package. The cluster parameter was set at 9 and the typing parameter was set at 2. Survival analysis was performed using survival R package and survminer R package based on genotyping results combined with clinical data retrieved from TCGA and GEO databases to explore the value of single genotype in TNBC (Wang et al., 2020). Heat maps were generated using pheatmap R package to show differences in TNBC patients with different genotypes. Boruta R package was used to analyze and identify characteristic genes based on genotyping results, and PCA (Principal Component Analysis) was performed to further explore the immune status. GO analysis (Gene Ontology analysis) was performed to explore biological processes of differentially expressed genes and KEGG analysis was conducted to identify possible pathways and results were presented as Sankey plots. GSEA (Gene Set Enrichment Analysis) was carried out based on the potential signaling pathways identified for further analysis of their effect in different groups.

### Mutation Analysis

Mutation data of TNBC patients were retrieved from TCGA database. A total of 115 cases of triple-negative breast cancer patients were screened out based on clinical information available in TCGA database. Tumor mutation burden (TMB) was determined based on the information of the 115 patients. TMB scores combined with genotype information and immune scores was used for correlation analysis across genotype, immune score and the TMB. Survival R package and survminer R package were used to explore prognosis of TMB high group and TMB low group based on TMB and immune scores (Zhang et al., 2020).

### Statistical Analysis

R software version 4.0.3 was used for all statistical analysis. Univariate and multivariate cox regression analysis were performed to evaluate survival situation. The hazard ratio (HR) and 95% confidence interval (CI) were calculated to identify genes related to overall survival. Except as otherwise noted, *p* < 0.05 was considered statistically significant.

## Results

### Immunophenotyping and Cluster Analysis

**FIGURE 2:**
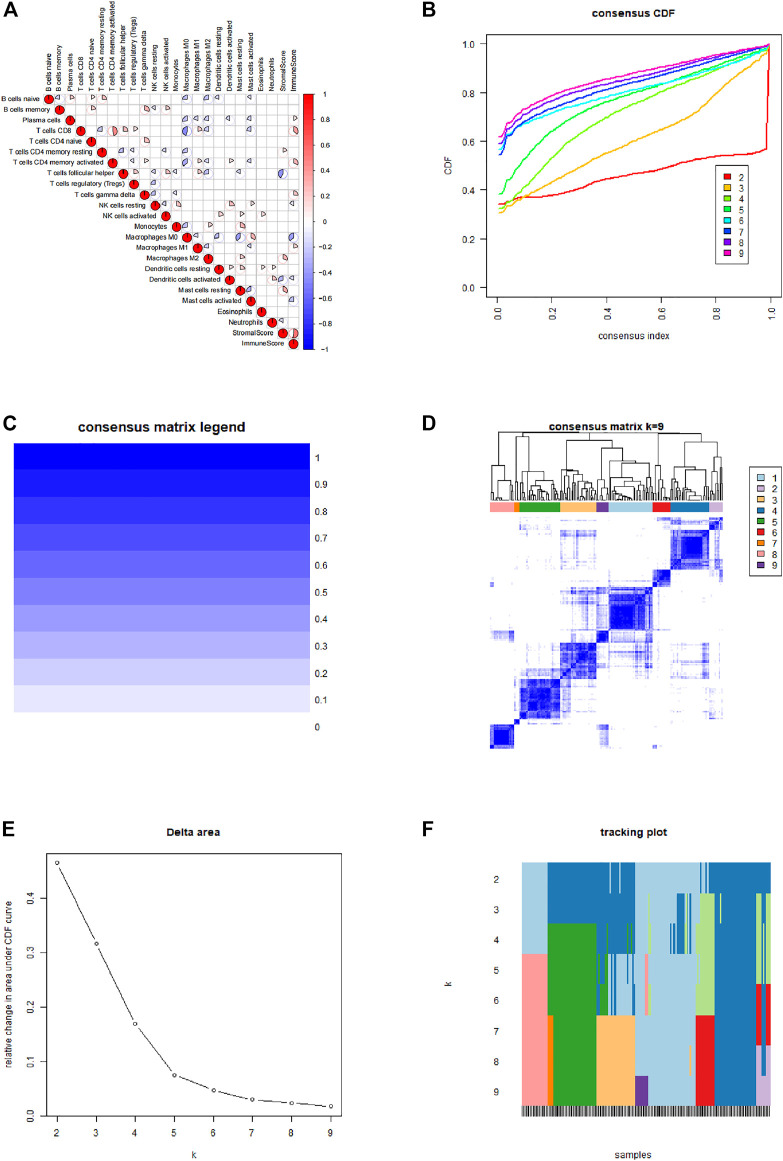
Immune typing and cluster analysis results. **(A)** Correlation between various types of immune infiltrating cells. **(B)** Cumulative distribution function of the cluster. **(C,D)** Cluster analysis matrix. **(E)** Delta area of the cluster analysis. **(F)** Tracking graph of cluster analysis.

After integrating TCGA and GEO data cluster analysis was performed using ConsensusClusterPlus (Figure 2). Correlation analysis was performed for the various types of immune infiltrating cells. The findings showed positive correlation between CD8 T cells and CD4 T cells activated memory cells. Analysis showed a negative correlation between Macrophages M0 and CD8 T cells. All patients were divided into 3 immune infiltration types, including group A, B, and C based on the clustering results, CIBERSORT results and the immune scores and matrix scores obtained from ESTIMATE.

### Prognostic Value of Immunophenotypes in TNBC

**FIGURE 3:**
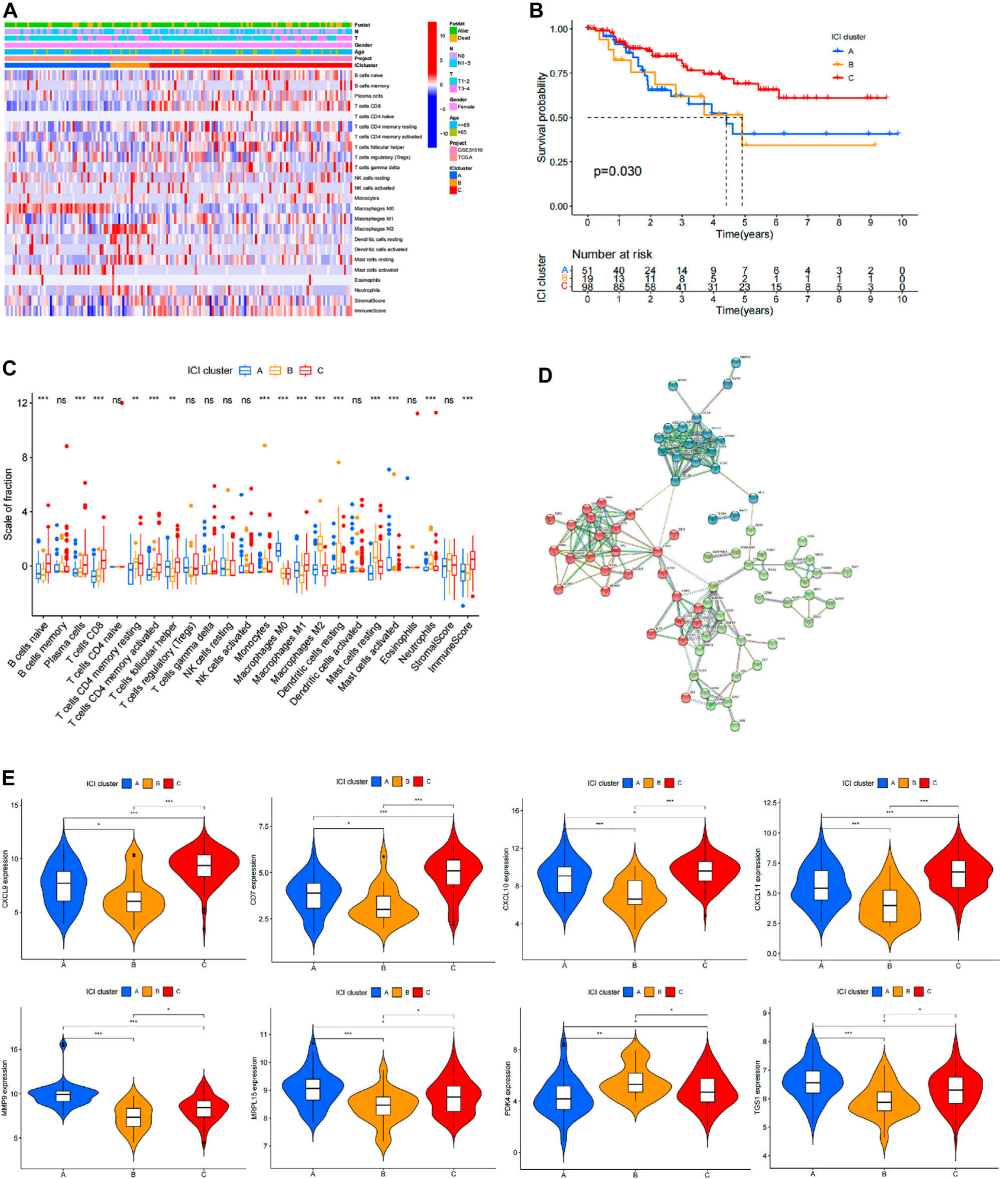
Prognostic value of immunophenotyping in TNBC patients. **(A)** Heat map showing immune cell infiltration, age, estimate score and patient information in different immune types. **(B)** Analysis of survival in different immune types. **(C)** Specific infiltration profile and differential analysis of the standard 22 immune cells in different immune types, where ✮ represents *p* < 0.05, ✮✮ represents *p* < 0.01, and ✮✮✮ represents *p* < 0.001. **(D)** STRING analysis results of genes that are differentially expressed in different immune types showing the correlation between these genes. **(E)** Expression levels of genes that are significantly differentially expressed in the three different immune types.

Basic information of the patient including age, TNM stage, survival information and situation of immune cell infiltration was presented as heat maps (Figure 3A). Survival analysis based on clinical information of TNBC shows significantly different prognosis between group B-C and group A-C (Figure 3B). Notably, prognostic analysis showed that survival of group A was not significantly different compared with that of group B. These findings indicate significant differences in prognosis of TNBC patients in different immune types, showing potential and value for evaluating prognosis of TNBC patients. Infiltration of 22 immune cells in different immune types were analyzed based on genes expression levels in different immune types and 22 standard immune cells using CIBERSORT R package, and T test was performed to compared the groups. A total of 15 immune cells including *B cells naive* and *Plasma cells* showed significant difference in different immune types, whereas 7 immune cells including *B cells* memory and *NK cells resting* showed no significant differences between different immune types (Figure 3C). In addition, the findings showed different infiltration levels of 15 immune cells in different immune types, indicating that infiltration of immune cells is can be used in immunophenotyping of different TNBC patients. Expression levels of genes in different immune types was compared by T-test analysis showed presence of differentially expressed genes in BC, AC, and AB. Analysis using STRING tool was performed to explore association of differentially expressed genes (Figure 3D). Differentially expressed genes in BC, AC, and AB showed intersection, and 8 genes significantly differentially expressed including CD7, CXCL9, CXCL10, CXCL11, MMP9, MRPL15, PDK4 and TGS1 were obtained from the intersection (Figure 3E). These genes were significantly differentially expressed in different immune types, and may be key regulatory factors for different prognosis in different immune types. Therefore, these genes are potential therapeutic targets and prognostic predictor markers.

### Role of Genotyping in Prognostic Analysis of TNBC

**FIGURE 4:**
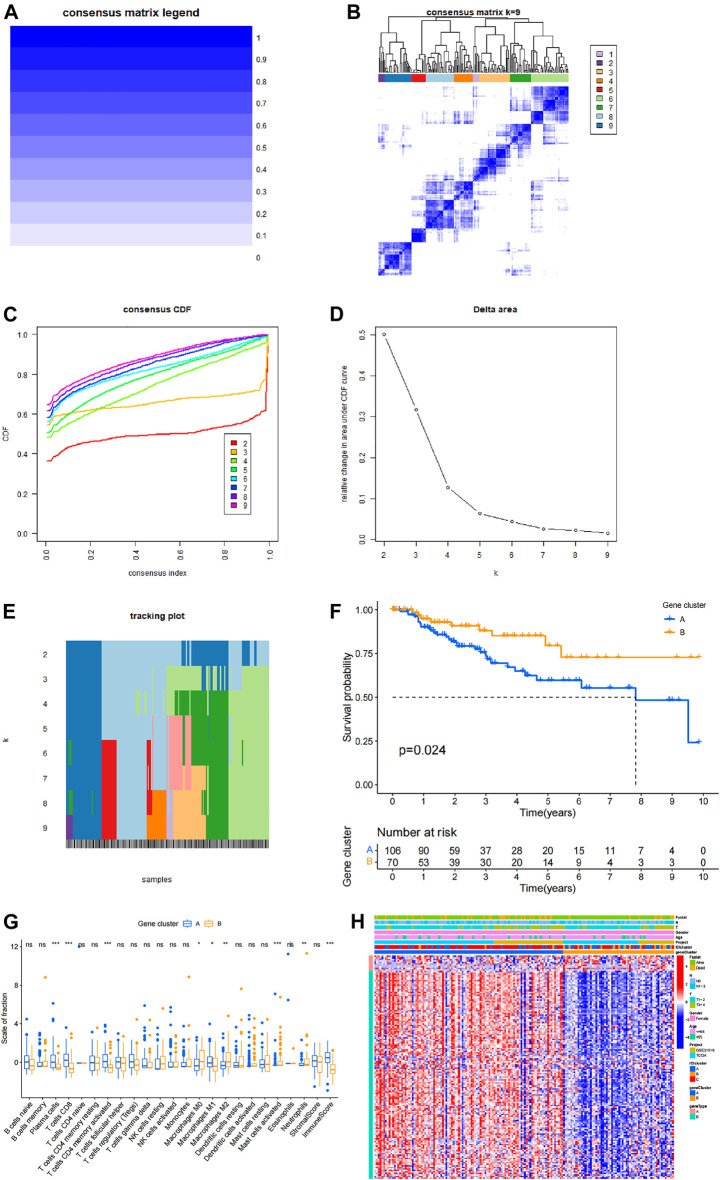
Prognostic value of genotyping in TNBC. **(A,B)** Cluster analysis matrices. **(C)** Cumulative distribution function of the cluster. **(D)** Delta area of the cluster analysis. **(E)** Tracking graph of cluster analysis. **(F)** Survival analysis based on genotyping. **(G)** Immune cell infiltration in different genotypes and analysis results, where ✮ represents *p* < 0.05, ✮✮ represents *p* < 0.01, ✮✮✮ represents *p* < 0.001. **(H)** A comprehensive heat map showing various information of patients in different genotypes and distribution of differentially infiltrated immune cells in each patient.

Cluster analysis based on differentially expressed genes in different immune type groups and expression data of all patients was performed using ConsensusClusterPlus R package (Figure 4). Survival analysis was performed based on clustering results and patient clinical information and the findings showed that the prognosis of patients with different gene types was significantly different (*p* = 0.024). These findings indicate that genotyping has great potential and value in assessing prognosis of TNBC patients. Correlation analysis was performed based on a combination of genotyping data and immune cell infiltration data. The findings showed that plasma cells, CD8 cells, CD4 memory activated T cells, M0 macrophages, M1 macrophages, M2 macrophages, activated mast cells and neutrophils had significantly different infiltration in different genotypes, whereas other immune cells did not show significant differences in different genotypes (Figure 4G). Information on patients in different genotypes and level of infiltrating cells, age and TMN stage are presented as a heat map in Figure 4H.

### Role of Comprehensive Immune Score in Prognostic Evaluation of TNBC Patients

**FIGURE 5:**
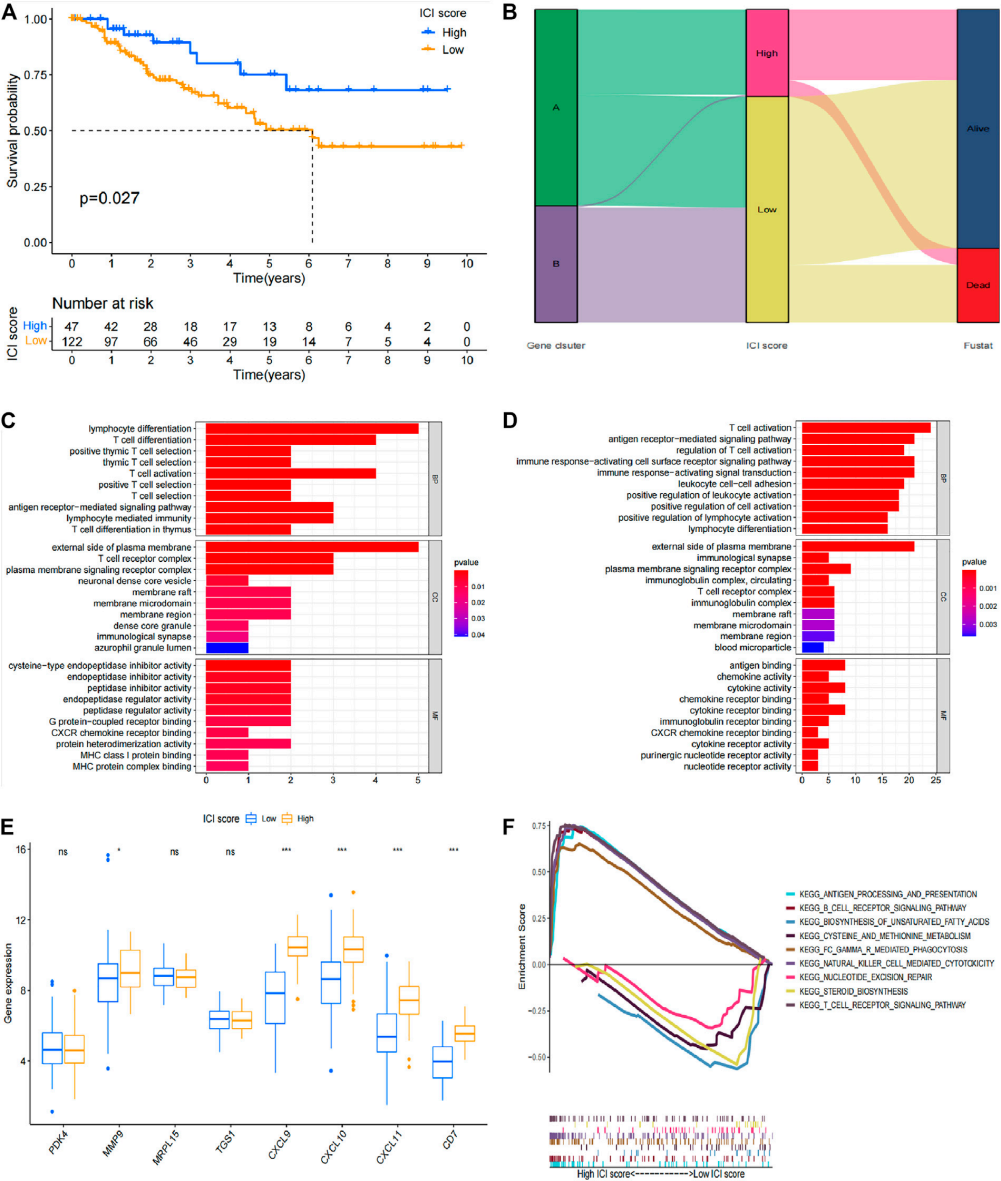
Prognostic value of immune score in TNBC patients. **(A)** Survival analysis results based on immune score. **(B)** Sankey diagram of immune score, genotyping and survival status. **(C)** GO analysis result for the high immune score group. **(D)** GO analysis results for the low immune score group. **(E)** Verification results of differentially expressed genes in different immunophenotypes and different immune score groups. **(F)** GSEA analysis results.

Boruta R package uses genotyping data to search for specific genes and performs principal component analysis (PCA). Immune scoring was performed on all patients based on these results and patients were divided into low immune score group and high immune score group based on the median value. Survival analysis was performed based on immune scoring results and clinical data of patients (Figure 5A). The high immune score group showed a better prognosis, which was significantly different from the prognosis of the low immune group (*p* = 0.027). The Sankey diagram in Figure 5 shows the relationship between genotyping, immune scores and prognosis of patients. The findings indicate that genotyping and immune scores have a significant impact on the prognosis of patients. GO analysis and GSEA analysis were conducted explore possible signaling pathways and to identify the functions of differentially expressed genes in different immune types. Signaling pathways with significant differentially enriched between high immune score and low immune score were screened through GO analysis (Figures 5C,D). Moreover, possible signaling pathways were verified through GSEA. The findings showed that antigen processing and presentation, B cell receptor signaling pathway, biosynthesis of unsaturated fatty acids, cysteine and methionine metabolism, FC gamma R mediated phagocytosis, natural killer cell mediating pathway, biosynthesis of unsaturated fatty acids, FC gamma R mediated phagocytosis, natural killer cell mediating pathway, biosynthesis of unsaturated fatty acids are possible signal pathways on comprehensive immune score (Figure 5F). The findings showed that MMP9, CXCL9, CXCL10, CXCL11 and CD7 were significantly differentially expressed in different immunotypes and different immune score groups (Figure 5E). These genes can be used for immune typing, immune score and prognosis evaluation of TNBC patients thus have significant potential for predicting prognosis of TNBC patients.

### Role of Mutation Score in Prognostic Analysis of TNBC Patients

**FIGURE 6:**
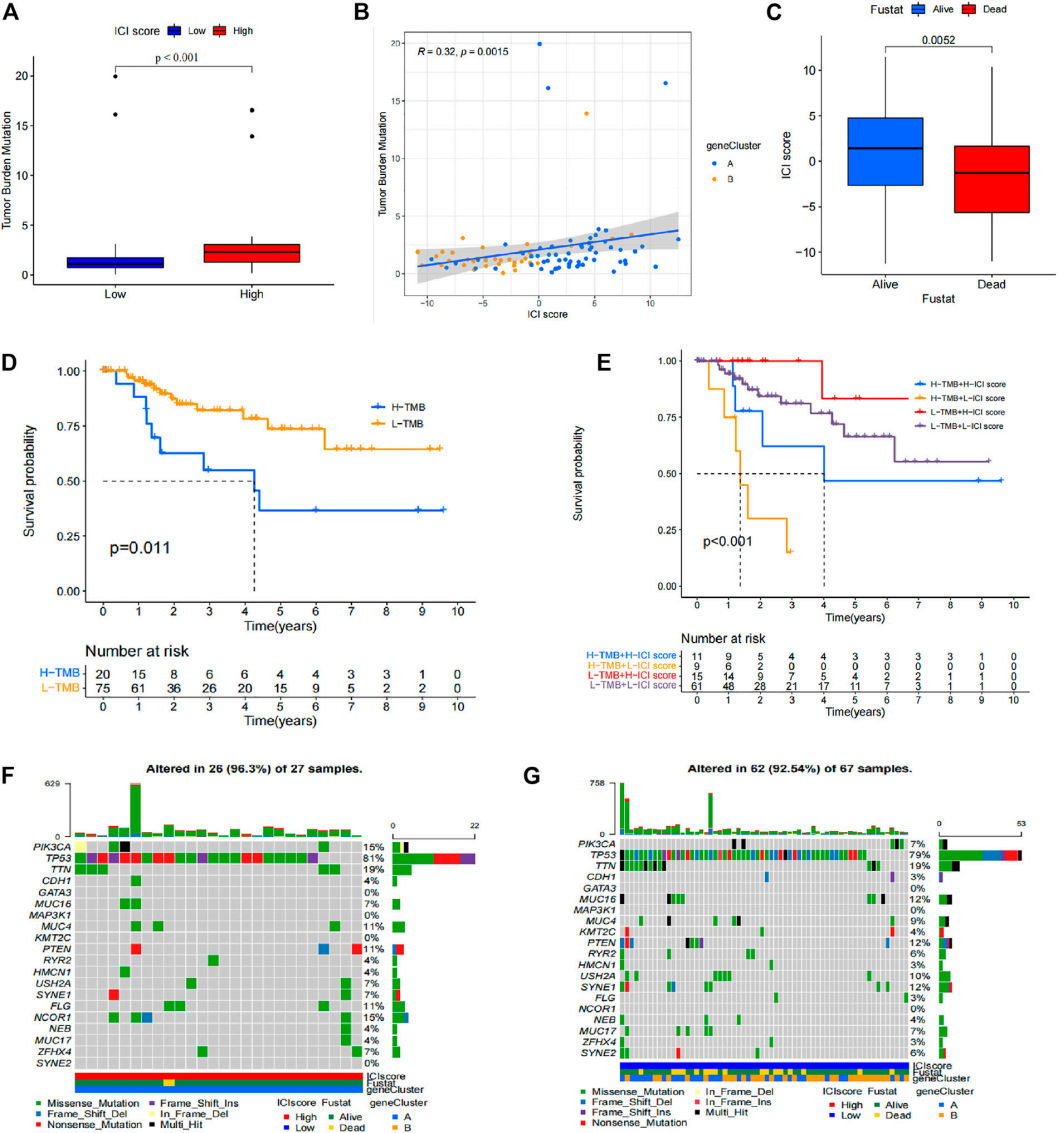
Prognostic value of mutation burden in TNBC. **(A)** Difference in mutation burden in patients with different immune scores. **(B)** Correlation between immune score and mutation burden. **(C)** Correlation between immune score and clinical information. **(D)** Different TMB prognostic analysis results. **(E)** TMB-immune score prognostic analysis results. **(F)** Waterfall chart of the high TMB group. **(G)** Waterfall chart of the low TMB group.

Mutation burden information was retrieved from TCGA database. The correlation between the mutation burden and the immune score was evaluated (Figures 6A,B), and the correlation between immune score and clinical information was also evaluated (Figure 6C). The findings showed that the high immune score group presented a high mutation burden, and a significant difference was observed in different score groups. These findings indicate that mutation burden is positively correlated with immune score. Survival analysis based on clinical information of patients showed that simple tumor mutation burden (TMB) was valuable for evaluation of patient prognosis. The high TMB group showed a poor prognosis compared with low TMB group (Figure 6D). Survival analysis was then performed using a combination of immune score and TMB data of patients. The findings showed that the low TMB with high immune score group had the best prognosis, whereas the high TMB with low immune score group showed the worst prognosis. The difference in prognosis between the groups was statistically significant (Figure 6E). Genes with significant mutations in different TMB groups was explored and the finding were expressed as a waterfall chart (Figures 6F,G). These findings indicated that these genes with significant mutation differences may play an important role in the different immune scores. However, further studies should be conducted to explore the mechanism.

## Discussion

TNBC is a breast cancer subtype with poor prognosis. Currently, there is no detailed classification available for accurate prognostic evaluation and effective treatment. Various tumor scores and immunotypes are based on immune cell infiltration in the tumor, has and have been used in liver cancer and are effective in evaluating prognosis of patients (Maecker et al., 2012; Cao et al., 2020). However, no studies have explored tumor scores in TNBC patients. Therefore, the current study performed immunophenotyping, genotyping, and mutation typing in TNBC patients based on data retrieved from TCGA and GEO databases, and by combining it with patient clinical information and mutation information. In addition, the prognostic value of these classification scores in TNBC patients and further possible mechanism were explored.

In immunophenotyping, patients were divided into three types based on ESTIMATE score and CIBERSORT score. Analysis showed that the high-infiltration group and the medium-infiltration group had significantly worse prognosis compared with prognosis of the low-infiltration group. The possible key genes include CD7, CXCL9, CXCL10, CXCL11, MMP9, MRPL15, PDK4 and TGS1. CD7 is the most sensitive antigen related to T-cells and is expressed in T-cell precursors, monocytes, and natural killer cells, related to various leukemia (Yu et al., 2017; Röhrs et al., 2010). CXCL9, CXCL10, CXCL11/CXCR3 axis has been proved to regulate immune cell migration, differentiation, and activation (Tokunaga et al., 2018; Neo et al., 2020), leading to tumor suppression in pancreatic adenocarcinoma, colorectal cancer and so on (Gao et al., 2020). MMP9 is a matricellular protein associated with extracellular matrix (ECM) remodelling, promoting tumour progression, and modulating the activity of cell adhesion molecules and cytokines (Joseph et al., 2020; Mondal et al., 2020). MRPL15 is a member of mitochondrial ribosomal proteins whose abnormal expression is related to tumorigenesis in lung cancer, epithelial ovarian cancer and so on (Xu et al., 2021; Zeng et al., 2021). PDK4 is a member of PDK family located in the mitochondrial matrix of cells, inhibiting the entry of pyruvate into the TCA cycle by inhibiting pyruvate dehydrogenase activity. PDK4 are highly up regulated in various cancers including glioblastoma, lung carcinoma, pancreatic cancer and breast cancer (Dixit et al., 2016; Guda et al., 2018; Tambe et al., 2019; Yu et al., 2021). TGS1 is a conserved enzyme that mediates formation of the trimethylguanosine cap on several RNAs, including snRNAs and telomerase RNA (Maccallini et al., 2020). But there is little research on TGS1 and cancer as yet. These genes may affect prognosis of TNBC patients by regulating infiltration of immune cells such as plasma cells, CD8 cells, CD4 memory activated T cells, M0 macrophages, M1 macrophages, M2 macrophages, activated mast cells and neutrophils. These key factors form a network for determining prognosis of TNBC patients.

In genotyping, patients were divided into two groups based on differentially expressed genes obtained from immunophenotyping and overall gene expression results. The two groups showed significant differences in prognosis, and the findings indicated that infiltration of immune cells plays a significant role prognosis of TNBC patients. Precision cancer medicine requires effective genotyping of every patient’s tumor to optimally design treatment plans (Meador et al., 2019), and the results of genotyping in TNBC are valuable in evaluating prognosis, and confirms that the key genes selected were valuable for prognostic evaluation of TNBC patients.

Immune score was evaluated based on the genotyping results resulting in a comprehensive immune typing. The findings showed a significant prognostic difference between the high immune group and the low immune group. GSEA analysis was performed to explore possible signaling pathways associated with selected genes. These signal pathways have not yet been experimentally verified, and further research is needed to explore the specific mechanisms that affect the immune score of TNBC patients. Immune score and mutation score were further combined to obtain comprehensive immune types, including high-immunity low-mutation group, high-immunity high-mutation group, low-immunity high-mutation group, and low-immunity low-mutation group. The findings showed that comprehensive immune typing is highly effective and accurate in assessing prognosis of TNBC patients. Analysis showed that MMP9, CXCL9, CXCL10, CXCL11 and CD7 are key genes that may affect immune typing of TNBC patients and play an important role in prediction of prognosis in TNBC patients.

## Conclusion

The current study presents an evaluation system based on immunophenotyping, which provides a more accurate prognostic evaluation tool for TNBC patients. Differentially expressed genes can be targeted to improve treatment of TNBC.

## Data Availability

The original contributions presented in the study are included in the article/Supplementary Material, further inquiries can be directed to the corresponding author.
